# Mucosal vaccination with enzymatically active *Helicobacter pylori γ*-glutamyl transferase (GGT) adjuvanted with STING-agonist elicits robust Th1/Th17 immunity in mice

**DOI:** 10.3389/fmicb.2026.1825914

**Published:** 2026-06-30

**Authors:** Monish Kumar, Jiayan Li, Navin Varadarajan

**Affiliations:** 1 Department of Chemical and Biomolecular Engineering, University of Houston, Houston, TX, United States; 2 Department of Biomedical Engineering, University of Houston, Houston, TX, United States

**Keywords:** *γ*-glutamyl transferase (GGT), *Helicobacter pylori*, mucosal vaccine, STING, Th17

## Abstract

*Helicobacter pylori* infection is the leading cause of chronic gastritis and a major risk factor for gastric cancer, yet no licensed vaccine is currently available. The *H. pylori γ*-glutamyl transferase (hpGGT) is a key virulence factor that contributes to bacterial colonization and persistence, making it a promising antigen for vaccine development. Although hpGGT has been reported to exert immunosuppressive effects through its enzymatic activity, whether the enzymatic activity affects its immunogenicity is unknown. To address this question, we intranasally immunized mice with recombinant wild-type hpGGT (WT-GGT) or a structurally similar, enzymatically inactive variant (In-GGT), each formulated with a liposomal STING-agonist adjuvant. Despite the potential for immunosuppression, the enzymatically active WT-GGT was the superior immunogen: it elicited robust Th1 and Th17 responses, along with strong gastric IgA production—immune features known to be critical for protection against *H. pylori*. These findings demonstrate that intranasal immunization with WT-GGT combined with a STING-agonist adjuvant induces mucosal and cellular immunity, supporting its potential as a viable vaccine strategy against *H. pylori*.

## Highlights

- The enzymatic activity of *H. pylori γ*-glutamyl transpeptidase (GGT) does not dampen immunogenicity when compared to inactive variant.
- GGT immunization with an intranasal STING agonist liposomal adjuvant drives Th1/Th17 T cell responses.
- Intranasal GGT immunization induces gastric IgA, reinforcing mucosal immunity.

## 1 Introduction

*Helicobacter pylori* (*H. pylori*) is a highly prevalent gastric pathogen infecting over 50% of the global population (Hooi et al., 2017). Chronic infection represents the primary risk factor for gastritis, peptic ulcers, and gastric cancer, which remains one of the leading causes of cancer-related mortality worldwide (Plummer et al., 2015; Thrift and El-Serag, 2020). Despite its clinical significance, there is currently no licensed vaccine *against H. pylori*, and antibiotic-based treatments are increasingly limited by the emergence of resistance (Savoldi et al., 2018). Over the past two decades, multiple vaccine strategies have been investigated, including urease, VacA, HpaA, Nap and CagA antigens formulated with adjuvants such as cholera toxin subunit B or alum (Zeng et al., 2015; Tobias et al., 2017; Li et al., 2014; Michetti et al., 1994; Yang et al., 2015). While some early-phase clinical trials have demonstrated partial efficacy, challenges persist, particularly in achieving durable protection at the mucosal interface (Sutton and Boag, 2019).

Multiple virulence factors like urease, CagA and VacA are implicated in the pathogenesis of *H. pylori* infection and have been explored as potential antigen candidates for vaccination. Gamma-glutamyl transpeptidase (GGT), a secreted and periplasmic enzyme, catalyzes the hydrolysis of glutamine to glutamate and ammonia, thereby facilitating bacterial survival within the acidic gastric niche (Oertli et al., 2013; Chevalier et al., 1999). Unlike other virulence factors such as urease or VacA, which mainly contribute to colonization or cytotoxicity, GGT exerts multifaceted roles that are directly linked to both bacterial persistence and disease progression. Specifically, GGT has been shown to impair T cell proliferation, induce apoptosis of gastric epithelial cells, and promote a tolerogenic environment that enables long-term infection (Chevalier et al., 1999; Schmees et al., 2007). Accumulating evidence also implicates GGT in the initiation of gastric carcinogenesis, as its metabolic byproducts contribute to DNA damage and epithelial transformation (Meng et al., 2021). These dual functions supporting bacterial adaptation and actively suppressing host immunity highlight GGT as an attractive vaccine candidate. Although GGT can serve as a vaccine candidate, the ability of its enzymatic activity (glutamine depletion) to induce immune tolerance by activating regulatory T cells (Treg) and inhibiting dendritic cells is a major concern.

In addition to antigen composition, the type and polarization of the immune response induced by a vaccine critically influence its effectiveness. In the context of *H. pylori* vaccination in humans, antibody titers do not reliably predict clearance or durable protection. In mouse studies, secretory IgA has been linked to reduced colonization and protection, but the relative contributions of IgA and IgG remain incompletely defined, and available human data suggest that antigen-specific antibodies more often reflect ongoing infection than durable immunity (Taylor et al., 2007; Czinn et al., 1993). By contrast, mucosal T cell responses, particularly Th1- and Th17-skewed CD4 T cell responses, appear to be more closely associated with protective outcomes in both preclinical and human challenge studies (Zeng et al., 2015; Aebischer et al., 2008). Reports suggesting protection against *H. pylori* even in the absence of B cells bolster the importance of T cell mediated immunity (Blanchard et al., 1999; Garhart et al., 2003). Th1 responses activate macrophages, skew antibodies to IgG2c, and suppress the Treg responses known to be induced by *H. pylori* (Rad et al., 2006). Th17 responses mediate infiltration of neutrophils to gastric mucosa, which further reduce bacterial burden (Flach et al., 2011; Dixon et al., 2016). In aggregate, a mucosal Th1/Th17 T cell response and secretory IgA are likely contributors in vaccine mediated protection of *H. pylori*.

Intranasal vaccination offers a promising approach for eliciting protective mucosal immunity, in contrast to parenteral routes that primarily induce systemic responses (Lycke, 2012). In this context, activation of the STING pathway has emerged as a potent strategy for driving mucosal and Th1/Th17-type immune responses (Corrales et al., 2015). In our previous work, we have reported on a liposomal STING agonist, NanoSTING, an adjuvant platform capable of enhancing antigen presentation and robust Th1/Th17 responses at mucosal sites (Leekha et al., 2024). In this study, we performed intranasal immunization with either the catalytically active or an engineered enzymatically inactive form of GGT with NanoSTING as the mucosal adjuvant. We systematically evaluated systemic and mucosal antibody responses and found that both formulations elicited serum IgG and IgA, with the enzymatically active (WT-GGT) vaccine achieving higher titers than the inactive (In-GGT) preparation. Flow cytometry further showed that both WT-GGT and In-GGT significantly increased the frequencies of Th1 (IFN-*γ*^+^) and Th17 (IL-17A^+^) CD4^+^ T cells, but the responses were stronger in WT-GGT immunized mice. Consistent with enhanced mucosal immunity at the site of *H. pylori* colonization, gastric homogenate supernatants from vaccinated mice contained elevated GGT-specific IgA. Collectively, these data indicate that mucosal immunization with the enzymatically active GGT generates a broad and strong systemic and mucosal response.

## 2 Materials and methods

### 2.1 Expression and purification of proteins

To express the WT-GGT protein, we cloned codon optimized gene block (IDT) encoding hpGGT with a 6 × His tag at N terminus and lacking the first 26 amino acid signal sequence in pET28a vector. To express In-GGT, we cloned the two subunits of hpGGT (27–379 and 381–567) in a pETDuet vector with the larger fragment bearing the 6 × His tag at N-terminus and the smaller fragment with the deletion of catalytic threonine.

To express the proteins, we transformed the resulting plasmids in *E. coli* BL21(DE3) cells and grew a single colony in Terrific Broth containing 50 μg/mL kanamycin at 37 °C. When the OD_600_ of the culture reached 0.5, we induced protein expression by adding 500 μM IPTG and let the culture grow for 8 h. We pelleted the bacteria and resuspended the pellet in ice cold lysis buffer (50 mM NaH_2_PO_4_, 300 mM NaCl, 25 mM imidazole, 0.1% Tween 20 and 5 U/mL universal nuclease (Pierce), pH = 8). We then lysed the bacteria using a French press and isolated the soluble fraction by centrifugation at 20,000 ×*g* for 30 min. The lysate was loaded on to a Ni-NTA chromatography column and subsequently washed with wash buffer (50 mM NaH_2_PO_4_, 300 mM NaCl, 25 mM imidazole, pH = 8). To remove the endotoxins, the column was washed with 200 column volumes of 0.1% Triton X-114 in DPBS. Triton X-114 was further washed using endotoxin free, 20 column volumes of DPBS. The protein was finally eluted in endotoxin free elution buffer (50 mM NaH_2_PO_4_, 300 mM NaCl, 500 mM imidazole, pH = 8). The protein was further purified using size exclusion chromatography (SEC) using Sephadex G-25 resin (Cytiva) and eluted in PBS. We measured the final endotoxin levels using chromogenic LAL endotoxin detection kit (Pierce) and endotoxin levels were <0.25 EU/mg of protein in all preparations.

### 2.2 NanoSTING preparation

We prepared cGAMP-encapsulating nanoparticles as described previously (Leekha et al., 2024). Briefly, we prepared a 10:1:1:1 mixture of DPPC, DPPG, Cholesterol (Chol), and DPPE-PEG2000 (Avanti Polar Lipids, AL, USA) in CHCl_3_ and CH_3_OH. The solvent was evaporated using a vacuum rotary evaporator at 45 °C to obtain a thin lipid film. We added prewarmed cGAMP (Medchem Express, NJ, USA) solution (1.5 mg/mL in PBS) to hydrate the film and sonicated the mixture for 60 min. The mixture was further subjected to five freeze–thaw cycles, and membrane extrusion through a 200 nm membrane (Cytiva, MA, USA, Cat #10417004) to generate particles of uniform size. Free, unencapsulated cGAMP was removed from the resulting nanoparticles using Amicon ultrafiltration units (10 kDa MW cutoff). We utilized an HPLC method to evaluate the encapsulation efficiency of cGAMP by generating a standard curve with cGAMP solutions ranging from 0.01 mg/mL to 0.15 mg/mL, followed by analysis using an HPLC column. To quantify the encapsulated cGAMP, liposomes were disrupted by adding methanol, releasing the cGAMP for analysis by HPLC. The resulting HPLC peak was then compared to the standard curve to determine the encapsulated cGAMP content.

### 2.3 SDS-PAGE

We analyzed the expressed and purified proteins by electrophoresing through 4–15% Mini-PROTEAN TGX (BioRad, CA, USA) gels under reducing and denaturing conditions. We added 2-mercaptoethanol (Sigma Life Science, Burlington, MA) to the protein samples to disrupt the disulfide bonds and incubated the samples at 95 °C for 5 min to denature the protein. Vertical electrophoresis was performed with 1 × Tris/glycine/SDS running buffer for 2 h at 90 V. To develop the gel, we used gel code blue stain (ThermoScientific) according to manufacturer’s protocol.

### 2.4 Mouse studies

We performed all mouse experiments in accordance with the guidelines provided by the Institutional Animal Care and Use Committee (IACUC). We purchased 6–10-week-old female BALB/c mice from The Jackson Laboratory (ME, USA). We housed them at the institutional animal facility of the University of Houston for at least 10 days before the start of the experiments. The animals were vaccinated intranasally using these vaccine formulations: 10 μg GGT + 1 μg Nano STING. Mice were anesthetized with isoflurane prior to intranasal vaccine administration. The vaccine was delivered at a volume of 10–15 μL per nostril, resulting in a total administered volume of 20–30 μL per mouse. Three weeks after the first dose, the mice were boosted with the corresponding prime dosage. We collected the blood via tail vein on day 21 and 45. All animals were euthanized 5 weeks after the first vaccination, and tissue (stomach and spleen) samples were collected for downstream analysis.

### 2.5 ELISA

We evaluated the magnitude of vaccine-induced antibody responses using both gastric tissue homogenates and serum samples. For gastric samples, we collected stomachs and 1 g of tissue was excised from each mouse. The tissue was cut into ~1 mm^3^ pieces and transferred into a 1.5 mL microcentrifuge tube containing 500 μL of T-PER™ tissue protein extraction reagent (Thermo Fisher Scientific, USA). A single stainless-steel bead was added to each tube, and homogenization was performed using a bead mill homogenizer until uniform lysates were obtained. Beads were removed, and the homogenates were centrifuged at 13,000 ×*g* for 15 min at 4 °C. Supernatants were carefully collected and used for detection of IgA using ELISA.

High protein-binding ELISA plates (Corning, NY, USA) were coated with GGT proteins at 0.5 μg/mL in phosphate-buffered saline (PBS) overnight at 4 °C or for 2 h at 37 °C. Plates were washed with PBS + 0.05% Tween-20 (PBST) to remove unbound proteins. Blocking conditions varied by sample type: serum samples were blocked with 1% BSA in PBS for 1 h at 37 °C, whereas gastric homogenate samples were blocked with 5% BSA in PBS for 2 h at 37 °C. After four additional washes with PBST, gastric homogenate supernatants or serum samples from immunized mice (Day 45) were added at serial dilutions. GGT protein–binding antibodies were detected using HRP-conjugated anti-mouse IgG (Jackson Immuno Research, 1:10000; PA, USA). For IgA detection, Goat HRP-conjugated anti-mouse IgA (Vector Laboratories, 1:15000; CA, USA) was applied. Plates were developed with 1-Step™ TMB ELISA substrate (Thermo Fisher Scientific) and absorbance was measured accordingly.

### 2.6 Splenocyte collection and lymphocyte intracellular cytokine staining

We harvested spleen cells from vaccinated mice to test antigen-specific T-cell responses. Spleens were collected in RPMI medium, then homogenized by passing through a 40 μm strainer. We removed the red blood cells by incubating the splenocytes with 3 mL of ACK lysis buffer for 3 min at RT. We washed the cells subsequently two times with RPMI 1640 medium containing 10% FBS. We resuspended the cells at a concentration of 2 million cells/ml. To study GGT specific responses we added heat inactivated WT-GGT (to abolish glutaminase activity) or In-GGT at a concentration of 5 μg/mL in a 24 well plate containing 2 million splenocytes for 3 days at 37 °C, 5% CO_2_.

Six hours before collecting the cells, we added Golgistop at 1 μM. For intracellular cytokine staining, we collected the activated splenocytes after 3 days of activation and washed them twice with PBS and stained the cells with BV510 aqua live/dead marker according to manufacturer’s protocol. To block the Fc receptors, we added CD16/CD32 blocking antibody (Clone 2.4G2, BD biosciences) to cells resuspended in FACS (2% FBS in PBS) buffer. The cells were then stained for extracellular markers CD3-BUV395 (BD Biosciences #569614), CD4-BV421 (Biolegend, #100543) and CD8-AF488 (Biolegend, #100726) at 4 °C for 1 h. The cells were then fixed for 20 min at 4 °C using BD cytofix solution, and subsequently washed two times with the cytoperm buffer (BD Biosciences). We then stained the cells with IFNγ-BV711 (Biolegend #505835) and IL17-PE (Biolegend #506903) in Cytoperm buffer overnight at 4 °C. We washed the cells two times with FACS buffer and read the cells at BD Fortessa flow cytometer.

### 2.7 *In-silico* prediction

We used Alphafold 3 server to predict the structures of WT-GGT and In-GGT. To align the protein structures and calculate the RMSD, we used ChimeraX (Abramson et al., 2024; Meng et al., 2023).

### 2.8 Multiple sequence alignment of GGT from different *H. pylori* strains

We obtained GGT sequences from genomic assembly of 1,006 strains from all over the world published in *Helicobacter pylori* genome project (BioProject: PRJNA529500) (Thorell et al., 2023). We performed multiple sequence alignment using Clustal Omega and calculated the normalized Shannon entropy for each residue using custom codes written in Python.

### 2.9 Statistical analysis

We presented all data as mean values, and error bars represent ± SEM (standard error of the mean). All statistical analyses were performed using GraphPad Prism (V10). We compared two groups using the Mann–Whitney U test, while comparisons between multiple groups were performed using Tukey’s multiple tests for repeated measures analysis.

## 3 Results

### 3.1 Expression and characterization of recombinant GGT antigens

**Figure 1:**
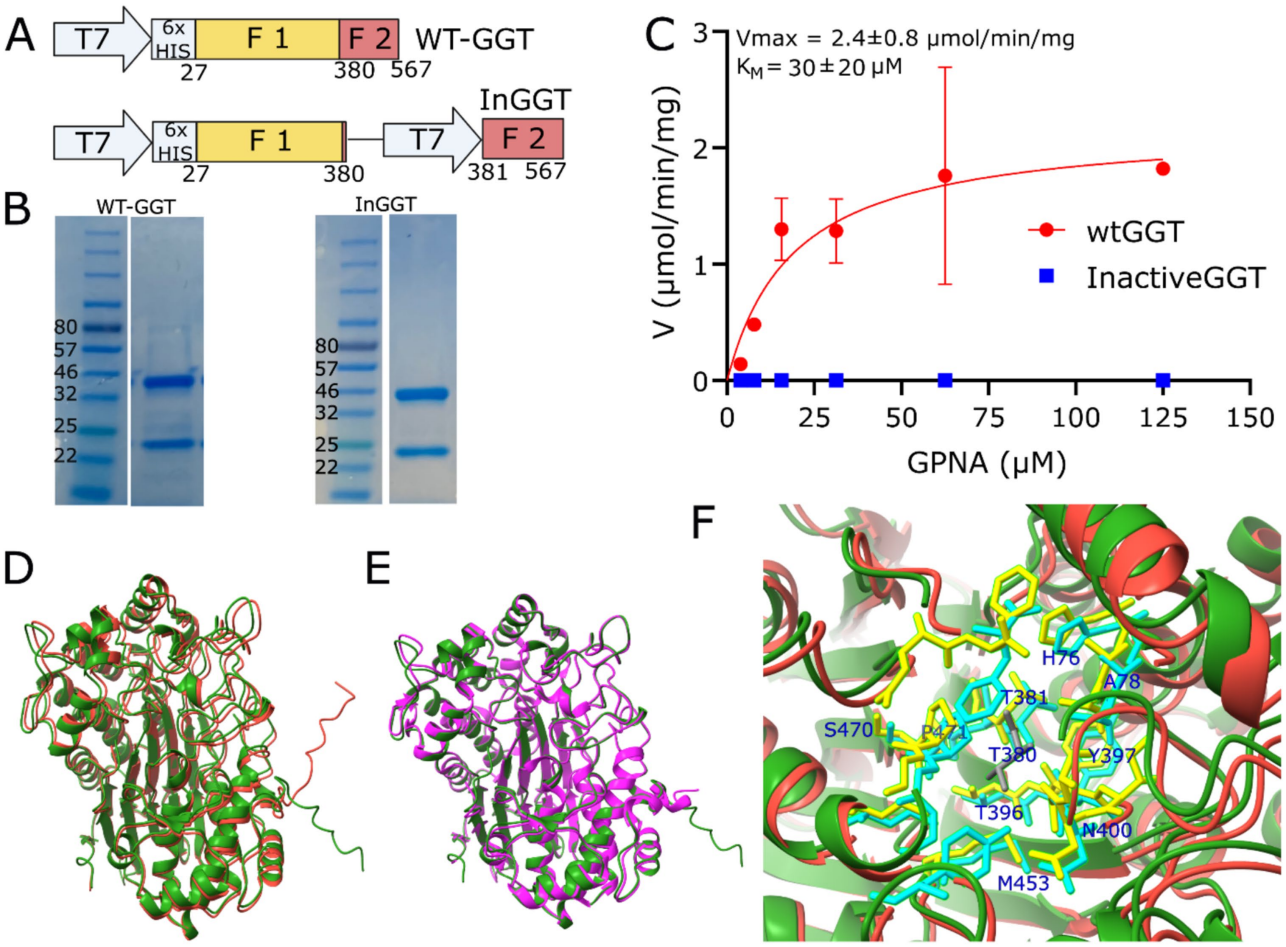
Expression and characterization of recombinant *H. pylori* wild-type GGT (WT-GGT) and inactive GGT (In-GGT). **(A)** Schematic of vector construct for expression of WT-GGT and In-GGT. **(B)** SDS PAGE analysis of purified WT-GGT (left panel) and In-GGT (right panel). **(C)** Plot showing catalytic activity of WT-GGT and In-GGT against GGT substrate GPNA. Dots represent the mean, and the error bars denote standard deviation. **(D)** Ribbon representation of predicted structures of WT-GGT and In-GGT. **(E)** Ribbon representation of aligned Alphafold 3 predicted structure of WT-GGT and experimentally determined structure of hpGGT via X-ray crystallography (PDB:2NQO). **(F)** Ball and stick representation of the aligned structure of WT-GGT and In-GGT in a 5 Å radius of the catalytic nucleophile T380 of the wild-type enzyme.

Wild-type *H. pylori γ*-glutamyl transpeptidase (hpGGT) is naturally expressed as a 60 kDa pro-peptide, which undergoes autoproteolysis generating two fragments of 40 kDa and 20 kDa held together by non-covalent interactions (Shibayama et al., 2003; Boanca et al., 2007). The N-terminal threonine of the smaller subunit acts as a nucleophile at the catalytic site. We expressed recombinant wild type *H. pylori γ*-glutamyl transpeptidase (WT-GGT) lacking the periplasm targeting signal sequence with an N-terminal hexa-histidine tag in *E. coli* BL21(DE3) cells (Figure 1A). We purified the protein using Ni-NTA based affinity chromatography followed by size exclusion chromatography. Consistent with autoproteolysis of WT-GGT peptide into two subunits, we observed two bands corresponding to the expected sizes in SDS-PAGE (Figure 1B) (Boanca et al., 2007; Shibayama et al., 2007). Using the chromogenic substrate *γ*-glutamyl paranitroaniline (GPNA), we confirmed that the WT-GGT enzyme was catalytically active with Michaelis–Menten kinetic constants in accordance with published values (V_max_ = 2.4 ± 0.8 μmol/min/mg and K_M_ = 30 ± 20 μM) (Boanca et al., 2007).

Since the primary enzymatic activity of hpGGT is to deplete glutamine and this in turn can lead to immunosuppression, we reasoned that a catalytically inactive variant that largely preserves the three-dimensional structure of the protein would act as a superior immunogen. Although mutating the catalytic threonine to alanine abolishes the enzymatic activity, it also abolishes autoproteolysis making it structurally unsuitable as an immunogen due to significantly different structure of the mature autoproteolyzed enzyme and the immature proenzyme (Boanca et al., 2006). Previous studies have reported that although the two subunits of hpGGT are insoluble when expressed individually, bicistronic expression leads to spontaneous heterodimerization and we used this approach to express the catalytically inactive variant (In-GGT, Figure 1A) (Boanca et al., 2006).

We expressed the protein in *E. coli* BL21(DE3) cells and purified the protein using affinity chromatography via hexa-histidine tag included in the N-terminus of the larger subunit. SDS-PAGE of the purified protein (Figure 1B) confirmed that we could detect two bands of 40 and 20 kDa even though the tag was only present on the larger subunit, an observation consistent with the self-assembly of the two subunits in the cytoplasm. Next, to confirm that In-GGT was catalytically inactive, we added the enzyme to GGT substrate *γ*-GPNA and confirmed that there was no hydrolysis of the substrate (Figure 1C).

We wanted to determine the structural similarity between In-GGT and WT-GGT. We used Alphafold 3 to predict the structures of WT-GGT and In-GGT (Figures 1D,E) (Abramson et al., 2024). When we modeled the two subunits in Alphafold 3, we obtained iPTM = 0.94 for both WT-GGT and In-GGT, indicating high-confidence inter-subunit orientation and a stable non-covalent interface, consistent with the known maturation of hpGGT into associated subunits. The root mean squared deviation (RMSD) of WT-GGT and In-GGT excluding the residues with low confidence prediction (pLDDT<50, residue 347–354 in WT-GGT) was 0.47 Å, indicating that the two structures were similar (Figure 1D). Furthermore, the RMSD of the two structures in a 5 Å radius of catalytic threonine T380 was 0.12 Å, indicating that the structure of catalytic site was preserved in In-GGT (Figure 1F). Collectively these results suggest that we expressed and purified a catalytically inactive variant, In-GGT, whose structure closely resembles that of WT-GGT.

### 3.2 Intranasal NanoSTING-GGT immunization induces systemic IgG and gastric IgA

**Figure 2:**
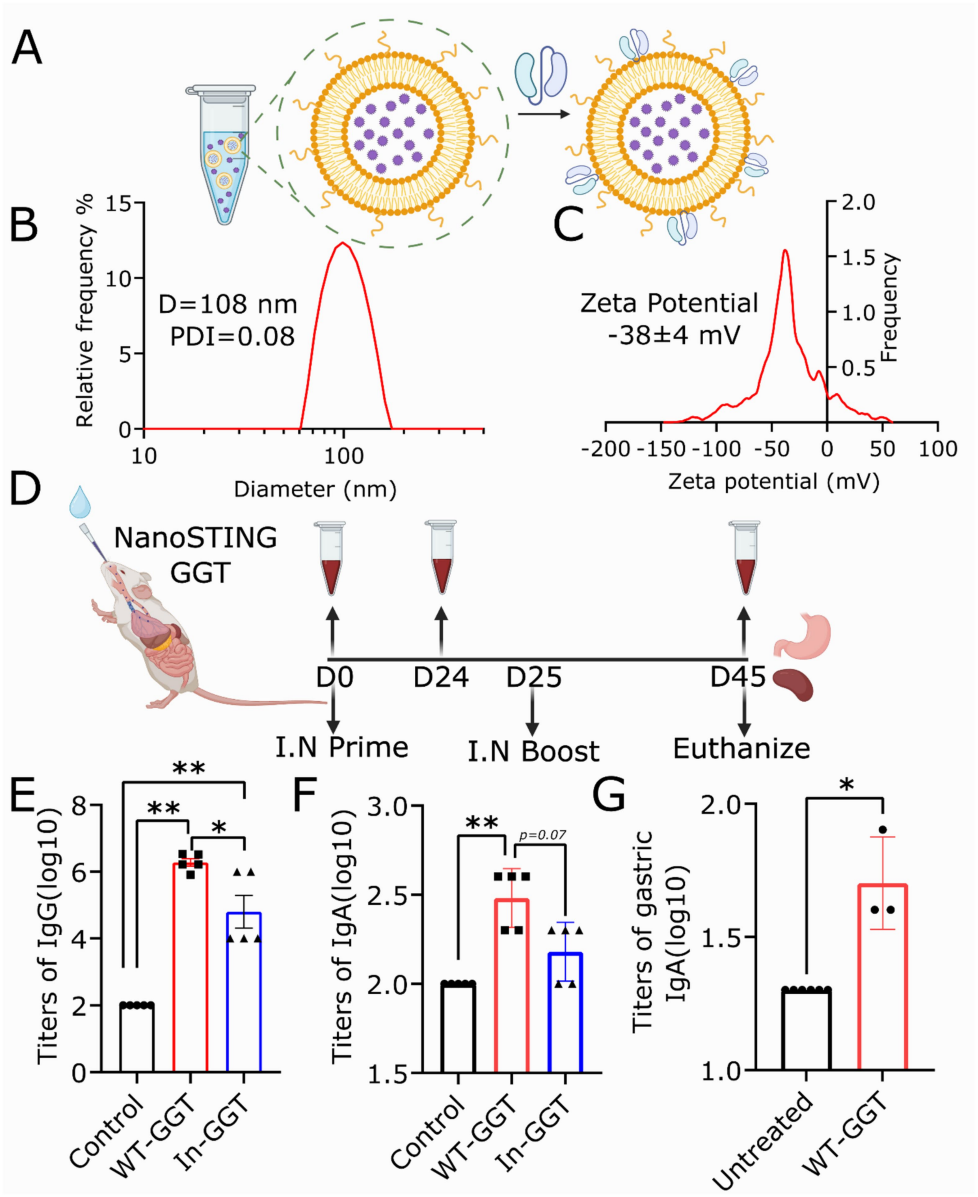
Intranasal immunization with NanoSTING and WT-GGT induces systemic and local IgG/IgA responses. **(A)** Schematic showing the vaccine formulation consisting of antigen and liposomes encapsulating 2′-3’ cGAMP. **(B,C)** Size distribution **(B)** and zeta-potential **(C)** of the liposomal adjuvant NanoSTING. **(D)** Schematic representing the immunization schedule and study design. **(E–F)** Serum **(E)** IgG and **(F)** IgA responses in mice immunized with WT-GGT and In-GGT. **(G)** Gastric IgA responses in mice immunized with WT-GGT. In (**E–G**) analysis was performed using a nonparametric test and vertical bars show mean values with error bars representing SEM. Mann–Whitney U test: ^∗∗∗^*p* < 0.001; ^∗∗^*p* < 0.01; ^∗^*p* < 0.05.

To assess the immunogenicity of the GGT antigens, we established an intranasal prime–boost vaccination schedule (Figure 2D) using a liposomal STING agonist (NanoSTING) as an adjuvant (Figure 2A). As we have previously demonstrated, the lipid composition in NanoSTING has been optimized for delivery in the respiratory tract (Leekha et al., 2024). Dynamic light scattering (DLS) analysis showed that the mean particle diameter of NanoSTING was 108 ± 31 nm (Figure 2B). Consistent with our prior publications, mean zeta potential of NanoSTING was −38 ± 4 mV (Figure 2C) (Leekha et al., 2024).

We dosed mice with 10 μg of WT-GGT or In-GGT formulated with 1 μg NanoSTING intranasally on Day 0 and Day 28. To assess systemic humoral immunity following intranasal vaccination, we evaluated the serum antibody titers by ELISA (Figures 2E,F). Immunization with WT-GGT induces strong antigen-specific serum IgG (inverse mean endpoint titer 2.0 ± 0.5 × 10^6^), while In-GGT induces lower titers (4 ± 2 × 10^5^) (Figure 2E). Contrary to our hypothesis that WT-GGT would dampen antigen induced immune responses, we observed higher IgG titers in animals treated with enzymatically active GGT compared to In-GGT (Figure 2E, *p*-value = 0.02).

Consistent with mucosal immunization, antigen-specific IgA were detected in the sera of WT-GGT vaccinated mice (3.2 ± 1.4 × 10^2^) and this was higher than the responses observed in In-GGT vaccinated mice (1.6 ± 0.7 × 10^2^, *p*-value = 0.07, Figure 2F). These findings demonstrate that WT-GGT-NanoSTING vaccination elicits robust systemic (IgG), and mucosal (IgA) humoral responses and these responses are superior to immunization with In-GGT-NanoSTING.

Since *H. pylori* establishes infection at the gastric mucosa, evaluating immune responses directly at this site provides the most physiologically relevant measure of protection. Measuring IgA in gastric homogenate supernatants therefore offers a direct assessment of local mucosal immunity, rather than relying solely on systemic readouts such as serum antibody titers, which may not accurately reflect protective capacity in the stomach (Czinn et al., 1993). Since animals immunized with WT-GGT-NanoSTING had significantly elevated serum IgA responses, we processed the gastric homogenates from these animals. Antigen-specific ELISA revealed, markedly elevated levels of GGT-specific IgA (mean endpoint titers 50 ± 30) in the vaccinated group, compared to controls (20 ± 0, *p*-value < 0.05; Figure 2G). These findings demonstrate that intranasal NanoSTING–GGT immunization elicits local mucosal IgA responses at the primary site of *H. pylori* colonization, underscoring the vaccine’s potential to support mucosal immunity.

### 3.3 Intranasal NanoSTING–GGT boosts Th1/Th17 CD4^+^ T cells

CD4^+^ T helper cells are central regulators of adaptive immunity and play critical roles in host defense against *H. pylori* (Oertli et al., 2013). On the one hand, CD4^+^ T helper cells provide indispensable help to B cells to generate protective antibodies that act directly in the stomach. On the other hand, CD4^+^ T cells differentiate into effector subsets—most notably Th17—that produce IL-17A/F and IL-22, which recruit neutrophils and induce epithelial antimicrobial programs that curb *H. pylori* bacterial load (Dixon et al., 2016). Given this dual role, assessing CD4^+^ T cell responses is a critical complement to antibody measurements, enabling a more comprehensive evaluation of vaccine-elicited protection.

**Figure 3:**
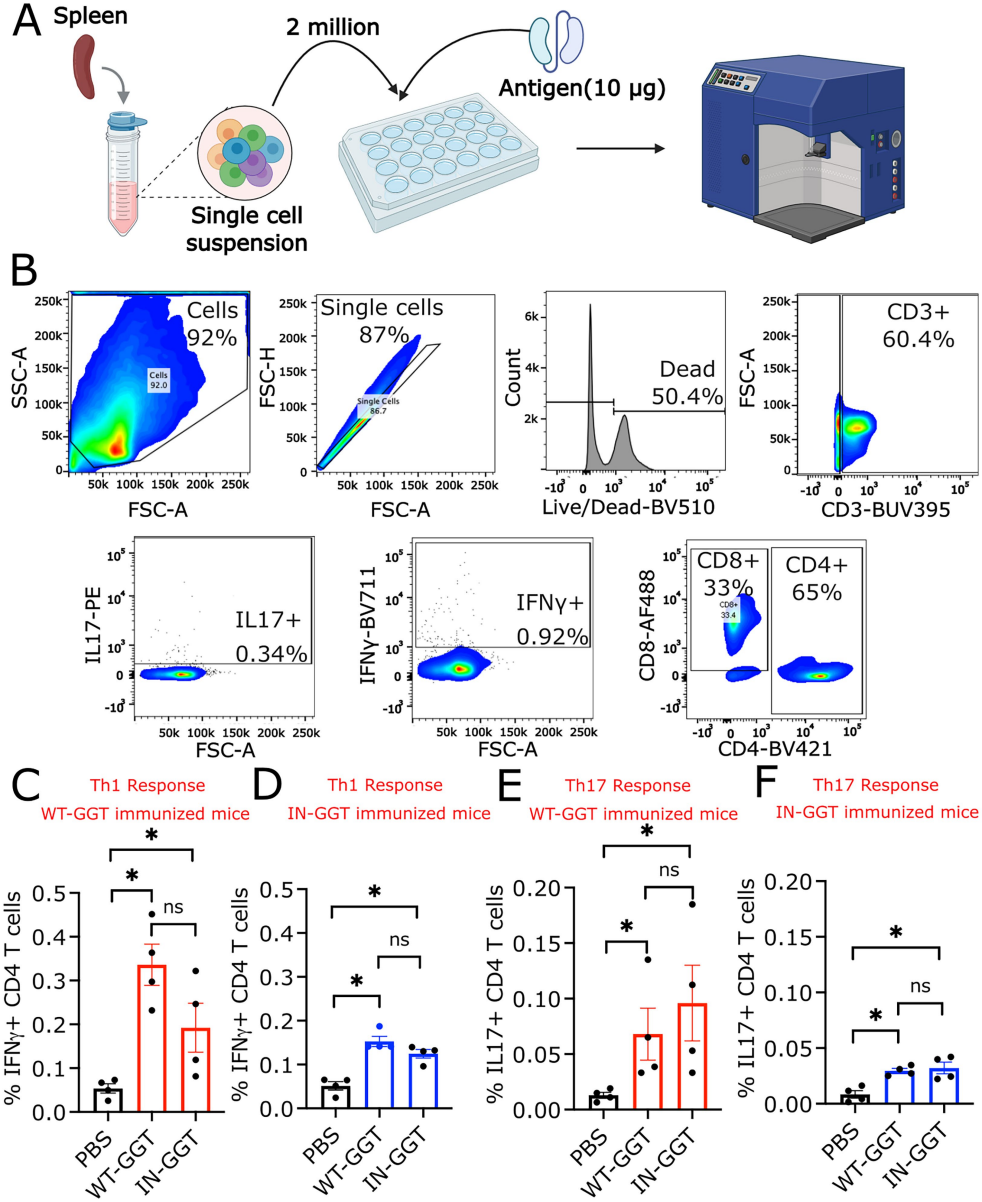
Intranasal immunization with NanoSTING and WT-GGT induces Th1/Th17 response. **(A)** Schematic showing the study design for assessing T cell responses upon immunization. **(B)** Gating strategy for detecting CD4^+^IFNγ/IL17^+^ T cells. (C,D) Frequency of CD4^+^IFNγ^+^ T cells upon **(C)** WT-GGT immunization and **(D)** In-GGT immunization measured using flow-cytometry. Labels on x-axis denotes the antigen used in stimulation. **(E,F)** Frequency of CD4^+^IL17^+^ T cells upon **(E)** WT-GGT and **(F)** In-GGT immunization. In **(C–F)** analysis was performed using a nonparametric test and vertical bars show mean values with error bars representing SEM. Mann–Whitney U test: ^∗∗∗^*p* < 0.001; ^∗∗^*p* < 0.01; ^∗^*p* < 0.05.

To this end, we collected splenocytes from immunized mice and treated the cells with 5 μg/mL of heat inactivated WT-GGT, In-GGT or PBS. We stimulated the splenocytes *ex vivo* with WT-GGT (WT-GGT stim) or In-GGT (In-GGT stim) to assess both direct and cross-reactive antigen responses in vaccinated mice (Figures 3A,B; Supplementary Figure S1A,B)). Mice immunized with WT-GGT showed significantly higher frequency of antigen-specific IFN-*γ*^+^ CD4^+^ T cells (WT-GGT stim: 0.35 ± 0.05, In-GGT stim: 0.20 ± 0.05, No stim: 0.05 ± 0.01) and IL17^+^ CD4^+^ Th17 T cells (WT-GGT stim: 0.07 ± 0.02, In-GGT stim: 0.09 ± 0.03, No stim: 0.01 ± 0.005) when compared to unstimulated media controls (Figures 3C,D; Supplementary Figure S2). Similarly, In-GGT immunized mice also showed significantly higher Th1 (WT-GGT stim: 0.15 ± 0.01, In-GGT stim: 0.12 ± 0.01, No stim: 0.05 ± 0.01) and Th17 responses (WT-GGT stim: 0.03 ± 0.002, In-GGT stim: 0.03 ± 0.005, No stim: 0.008 ± 0.002) when compared to unstimulated controls (Figures 3E,F; Supplementary Figure S2). The magnitude of both the Th1 and Th17 responses was superior in the splenocytes of WT-GGT immunized mice compared to the In-GGT immunized mice, regardless of the antigen used for *ex vivo* stimulation (Supplementary Figure S1C,E). Since the magnitude of the Th1/Th17 responses elicited in WT-GGT immunized mice in response to *ex vivo* activation with In-GGT was comparable if not superior to the responses of In-GGT immunized mice in response to *ex vivo* activation with In-GGT, we conclude that immunization with WT-GGT induces a strong Th1/Th17 response which is more potent than immunization with In-GGT (Supplementary Figure S1D,F). These observations are consistent with our observation of stronger antibody responses in WT-GGT immunized mice. Collectively, these findings demonstrate that intranasal NanoSTING–WT-GGT vaccination promotes strong effector T helper cell responses, reinforcing both humoral and cellular arms of immunity against *H. pylori*.

## 4 Discussion

Gastric cancer is the fifth leading cause of cancer-related mortality worldwide (Bray et al., 2024). Without effective interventions, approximately 15.6 million cases of gastric cancer are projected among individuals born between 2008 and 2017, with *Helicobacter pylori* infection accounting for an estimated 76% of these cases (Park et al., 2025). Meta-analyses of randomized controlled trials and observational studies consistently demonstrate that eradication of *H. pylori* infection in seropositive individuals significantly reduces gastric cancer risk, regardless of study design (Ford et al., 2025). Although *H. pylori* can be treated using combinations of antibiotics and proton pump inhibitors, the growing prevalence of antibiotic resistance poses a major challenge to long-term disease control. Vaccination therefore represents a promising strategy not just to prevent *H. pylori* infection but also minimize the associated pathologies. To date, vaccine development efforts have targeted multiple epitopes from key virulence factors including Urease, CagA, VacA, Nap, and *γ*-GGT delivered via intramuscular, oral, or intranasal routes (Sutton and Boag, 2019). However, no *H. pylori* vaccine has yet been approved for prophylactic or therapeutic use.

In this study, we aimed to evaluate the immunogenicity of γ-GGT utilizing our liposomal STING agonist platform as a mucosal adjuvant. We selected *Helicobacter pylori* γ-glutamyl transpeptidase (hpGGT) as the target antigen due to its critical role in bacterial colonization and persistence (Oertli et al., 2013; Chevalier et al., 1999). *H. pylori* mutants lacking hpGGT are unable to colonize the stomachs of neonatal mice and fail to persist in adult mice, with both effects dependent on the enzyme’s catalytic activity (Oertli et al., 2013). Through its hydrolase function, hpGGT depletes glutamine, thereby impairing dendritic cell and T cell activity and dampening the host immune response (Schmees et al., 2007; Müller et al., 2011). In addition to its key functional roles in immune invasion, one of the attractive features of hpGGT is that unlike sequences of VacA, the sequence tends to be highly conserved (Lai et al., 2025). Our analysis of hpGGT sequences across 1,006 clinically reported strains across the globe showed that its sequence is highly conserved, with 563 of 568 residues having a normalized Shannon entropy of <0.2 indicating strong conservation (Supplementary Figure S3) (Thorell et al., 2023). We hypothesized that targeting hpGGT, an enzyme essential for bacterial survival could serve as a promising vaccination strategy. Although an intranasal vaccine combining HspA and hpGGT has previously been reported, the antigen used included only the smaller subunit of hpGGT and lacked the catalytic pocket, failing to capture the full structural complexity of the enzyme (Zhang et al., 2015).

Because hpGGT enzymatic activity has been linked to immunosuppression, we investigated whether *H. pylori* GGT activity influences vaccine-induced immune responses. To this end, we generated a catalytically inactive variant of hpGGT (In-GGT) that retains the structural integrity of the mature wild-type enzyme (RMSD <1 Å). Upon immunization of mice with either wild-type GGT (WT-GGT) or In-GGT, the WT-GGT group exhibited significantly higher immunoglobulin (IgG, IgA) titers and stronger T cell responses.

There are several hypotheses that can explain the superior immunogenicity of WT-GGT compared to In-GGT. First, although both modeling and protein expression/purification data support the spontaneous assembly of the F1 and F2 subunits of In-GGT, consistent with previous reports, the stability and half-life of the In-GGT are not known (Boanca et al., 2006). The stability of proteins can alter the kinetics of proteolytic degradation during uptake and subsequent processing by antigen presenting cells (APCs) (Thai et al., 2004; Scheiblhofer et al., 2017). This alteration in kinetics determines the availability of antigenic peptides for MHC class II loading and presentation, and, thus directly dampens T cell responses (Fazilleau et al., 2009; Machado et al., 2016; Delamarre et al., 2006). Similarly, the stability of the folded protein also impacts conformational epitopes accessible to antibodies and B-cell mediated responses, and stabilization of conformations is routinely used in vaccine design to elicit neutralizing antibodies (Joyce et al., 2016; Langedijk et al., 2024). Consistent with this hypothesis, our immunogenicity data support that In-GGT had both lower T and B cell responses after immunization. A second and not necessarily exclusive hypothesis is that the immunogenicity might be related to the enzyme’s catalytic activity on cysteinyl leukotriene LTC_4_. WT-GGT would convert LTC_4_ to LTD_4_, a more potent CysLT1 antagonist, which is known to promote dendritic cell migration to lymph nodes in mice (Robbiani et al., 2000). We emphasize however that regardless of the underlying reason, our data conclusively demonstrate that WT-GGT immunized with NanoSTING as a mucosal adjuvant facilitates a strong immune response characterized by systemic IgG, mucosal IgA, and both Th1/Th17 T cell responses.

Given its multifaceted roles in bacterial persistence and colonization, hpGGT represents a strong vaccine candidate. Although hpGGT has been associated with immunosuppressive effects, our results demonstrate that these concerns are not relevant in murine immunization models. Using hpGGT in combination with a STING agonist, our immunization platform elicited robust local and systemic antibody responses against hpGGT. Consistent with previous studies highlighting the importance of Th1/Th17 polarization for protective immunity against *H. pylori*, our vaccine effectively induced these responses while also generating strong gastric IgA production—a hallmark of mucosal protection. While our hpGGT-NanoSTING vaccine induces strong immunogenicity, future studies involving challenge models with *H. pylori* should be undertaken to determine if the vaccine induced immune responses protect against either *H. pylori* infection prophylactically or can help control the spread of infection when administered as a therapeutic vaccine.

Preventing *H. pylori* infections via vaccination has proven to be extremely challenging (Duan et al., 2025). One of the major advantages of directly targeting GGT through immunization is that it provides an opportunity to directly interrupt the GGT induced biochemical microenvironment that facilitates gastric oncogenesis, even if the vaccine cannot prevent infections (Jiang et al., 2024). GGT contributes to the destruction of gastric mucosa via its primary function of glutamine depletion and the generation of hydrogen peroxide (Jiang et al., 2024). These metabolic and biochemical changes set off a cascade that triggers activation of the NF-κB pathway, release of interleukin-8, and recruitment of neutrophils culminating in chronic gastritis (Wüstner et al., 2017). Chronic gastritis is the first step in the widely accepted Correa pathway model describing the histological progression of intestinal-type gastric cancer (Li and Zhang, 2025). Immunological targeting of GGT through vaccination can thus provide an avenue to interrupting the ability of *H. pylori* to promote gastric cancer.

## Data Availability

The original contributions presented in the study are included in the article/Supplementary material, further inquiries can be directed to the corresponding author.
