# Supplementary material for: Mucosal vaccination with enzymatically active Helicobacter pylori γ-glutamyl transferase (GGT) adjuvanted with STING-agonist elicits robust Th1/Th17 immunity in mice
Source: Front Microbiol. 2026 Jun 30;17:1825914. doi: 10.3389/fmicb.2026.1825914 (PMC13365029; doi:10.3389/fmicb.2026.1825914)
Supplement: Supplementary file 1 [file Supplementary_file_1.docx]

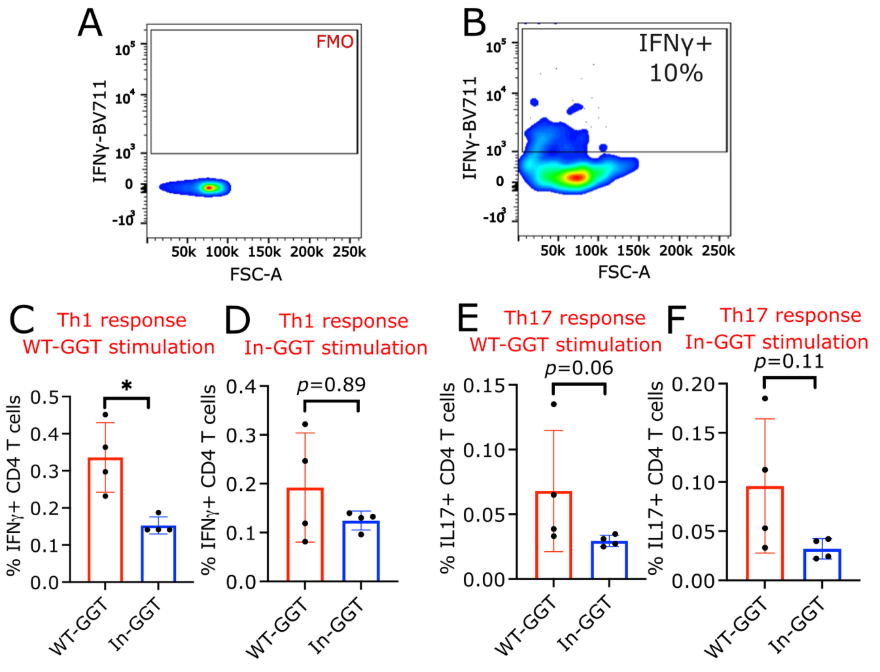


**Figure S1. Intranasal immunization with NanoSTING and WT-GGT induces Th1/Th17 response**

(A) Representative flow-cytometry plot showing FMO staining for IFNγ.

(B) Representative flow-cytometry plot showing IFNγ response in splenocytes upon PMA/Ionomycin stimulation.

(C,E) (C) Th1 and (E)Th17 responses upon stimulating with WT-GGT antigen in mice immunized with WT-GGT and In-GGT.

(D,F) (D) Th1 and (F)Th17 responses upon stimulating with In-GGT antigen in mice immunized with WT-GGT and In-GGT.

*In C, D, E, and F, analysis was performed using nonparametric test and vertical bars show mean values with error bars representing SEM. Mann-Whitney U test: ∗∗∗p < 0.001; ∗∗p < 0.01; ∗p < 0.05.*


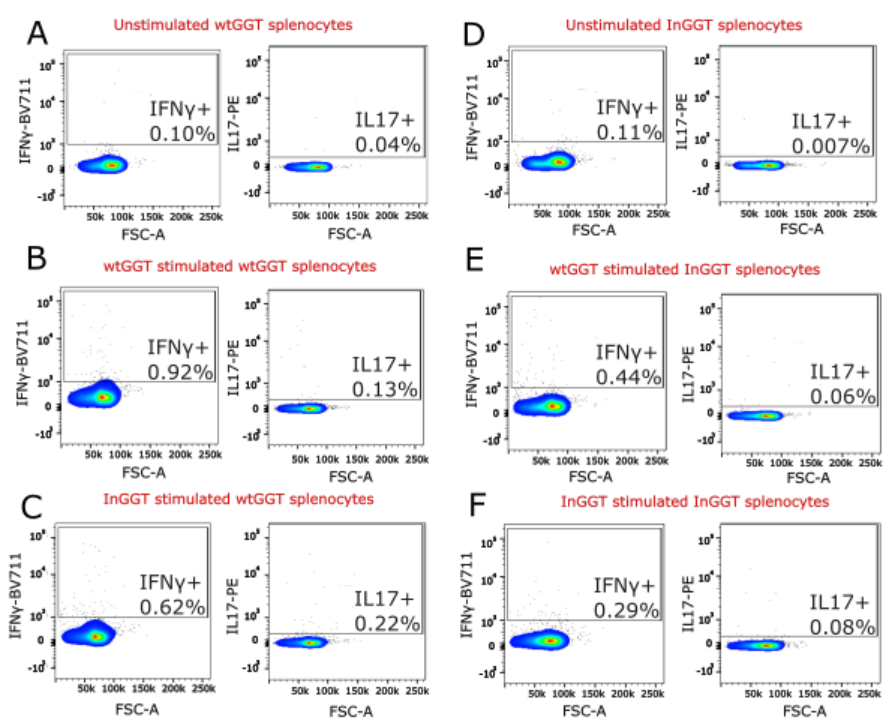


**Figure S2. Representative flow plots of Th1/Th17 responses**

(A,B,C) Splenocytes from mice immunized with WT-GGT and (A) no *ex vivo* stimulation (media control), (B) stimulated with WT-GGT protein, and (C) stimulated with In-GGT protein.

(D,E,F) Splenocytes from mice immunized with In-GGT and (A) no *ex vivo* stimulation (media control), (B) stimulated with WT-GGT protein, and (C) stimulated with In-GGT protein.


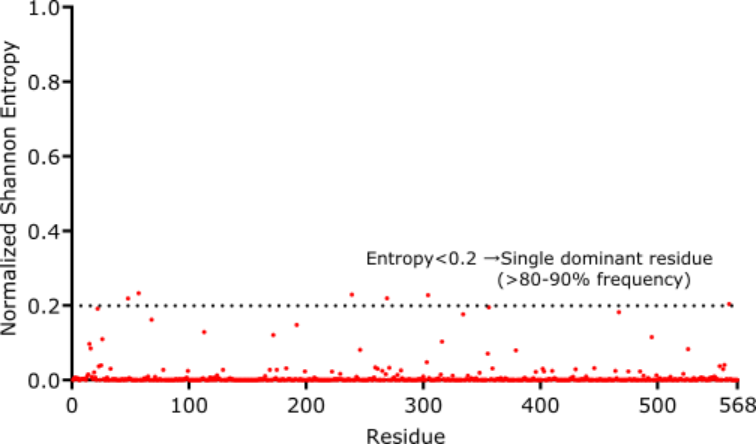


**Figure S3. Shannon entropy plot showing conservation of hpGGT among 1006 strains of *H. pylori*.** The GGT sequences were obtained from genomic assembly of 1006 strains from all over the world published in *Helicobacter pylori* genome project (BioProject: PRJNA529500)
